# Hiding in Plain Sight: Cell Biomimicry for Improving Hematological Cancer Outcomes

**DOI:** 10.3390/nano15100739

**Published:** 2025-05-15

**Authors:** Laura A. Weinstein, Bingqing Wei

**Affiliations:** 1 Department of Biomedical Engineering, University of Delaware, Newark 19716, DE, USA; lawny@udel.edu; 2 Department of Mechanical Engineering, University of Delaware, Newark 19716, DE, USA

**Keywords:** nanomedicine, biomimicry, drug delivery, cancer, homotypic targeting

## Abstract

The field of nanomedicine has been fruitful in creating novel drug delivery ideas to battle hematologic cancers. However, one persistent barrier to efficient nanoparticle treatment is phagocytic uptake or the clearance of nanoparticles by immune cells. To prevent this immune uptake, scientists have utilized biomimicry, the emulation of natural structures for engineered applications, to create particles that are able to remain unrecognized by immune cells. This method aims to improve the overall circulation time of nanoparticles by decreasing the amount of particles filtered out of the blood. It can even lead to homotypic cancer cell targeting, decreasing cancer cell vitality. This review summarizes recent in vivo and in vitro studies to prove that biomimetic cargo delivery is a unique and tenable way of increasing survival outcomes in patients with hematologic cancers.

## 1. Introduction

**Figure 1:**
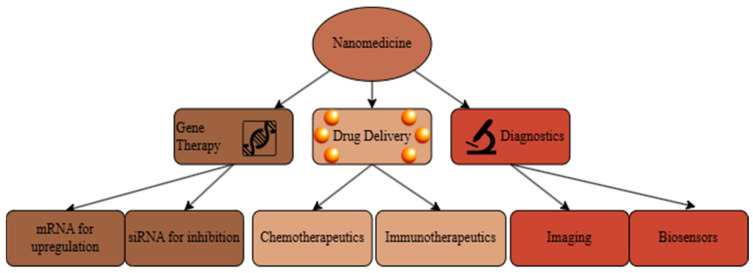
Flow chart of modern research avenues in nanomedicine, adapted from [8]. It includes fields of gene therapy, drug delivery, and diagnostics.

Nanoparticles, defined as materials between 1 and 100 nanometers in size, have a wide array of uses in medicine [1]. As schematically shown in Figure 1, this aptly named nanomedicine subfield covers a large amount of ground within the science of human health. Nanomedicines range from tunable gold shells used for photothermal therapy to transfection agents for gene delivery to the new field of theranostics that combines treatment and diagnosis [2]. At its core, nanoparticles are medicinal suitcases that allow for the apt delivery of diagnostic tools, genes, and more. Researchers today also investigate nanoparticles as preventative medicine tools by creating personalized vaccines made of nanoscale particles [3]. However, most of today’s literature focuses on nanomedicine as a tool for prolonged drug delivery [4,5,6]. Recent advances in nanoparticle-based drug delivery systems have revolutionized the landscape of disease treatment, offering enhanced therapeutic efficacy and reduced side effects. These systems are precisely engineered for controlled release, improved biocompatibility, and targeted drug delivery [7].

Currently, a deluge of research is being completed on nanomedicine as a cure for hematological cancers [9]. Cancer, in general, is one of the most challenging diseases to conquer; even the best therapies we have today, such as chemotherapy, surgery, radiation, and combinations of the former, have devastating side effects [10]. Unlike other cancers, hematological cancers affect the blood and its cells, such as leukemia, lymphoma, and multiple myeloma [11]. They occur within the body’s vasculature instead of within organ tissue and do not tend to form solid tumors [12].

Leukemia is the cancerous proliferation of hematopoietic stem cells within bone marrow [13]. In lymphoma, traditionally categorized as either Hodgkins (Hs) or Non-Hodgkins (NHs), lymphoid progenitor cells create mutated Reed-Sternberg cells (Hs) or mutated NK, T, or B cells (NHs) [14]. Multiple myeloma involves the mutation and rapid growth of plasma cells in the bone marrow [15]. Traditional methods of treating these malignancies include chemotherapies and hematopoietic stem cell transplants. However, these require high-dose chemotherapies that have toxic side effects and preparative conditioning therapy for transplants, which destroys both cancer and healthy cells [16,17].

In the United States, approximately every three minutes, someone is diagnosed with blood cancer, and every 9 min, someone dies from blood cancer [11]. In addition, hematological cancers also appear as side effects of other diseases, including human immunodeficiency virus, which vastly increases morbidity and mortality rates [18,19]. Therefore, finding a stable therapeutic for treating hematologic cancers is vital in today’s medical landscape.

One of the grand challenges in nanomedicine is finding a way to prolong the circulation time of nanoparticles in the bloodstream [20]. As soon as particles enter the body, they are assaulted by a flood of proteins that label the nanoparticles as foreign objects meant for immune clearance, which is known as opsonization [21]. Through opsonization, nanoparticles are rapidly filtered by means of the reticuloendothelial system, rendering them wholly ineffective at delivering their drug cargo to a target cancer outside the liver [22].

When particles are able to bypass this system, though, nanoparticles have many ways of entering solid tumors. The enhanced permeability and retention effect (EPR) explains how the leakiness of tumor vasculature allows for increased passive targeting [23]. Combined with the active passage of nanoparticles through the transendothelial barrier [24], the physiological alterations caused by solid tumors make their own gateways for nanoparticle treatment. Although not easy in any sense, solid tumors have physiological markers, such as an abnormally acidic microenvironment [25], that make them capable of targeting by current nanomedicines. Blood cancers, however, have no such apparent environment that allows for direct targeting [25]. In addition, the entire human body uses the bloodstream for oxygen and nutrients, which makes the stakes much higher for a drug delivery system; if a chemotherapeutic delivery were erroneously delivered in the bloodstream, this could have dire effects on any downstream tissues [26]. As such, “targeting” in terms of hematological cancers refers to the efficiency with which a particle can enter a cancer cell if it is able to stay in circulation long enough to encounter one [27].

Overall, for nanomedicines to be effective for hematological cancers, they need two essential components: (1) prolonged circulation within the circulatory system [28] and (2) effective and targeted drug delivery [29]. Herein, biomimicry is a proposed solution to both of these prevalent issues, as evidenced and summarized in this review.

## 2. Methods of Nanoparticle Shielding

**Figure 2:**
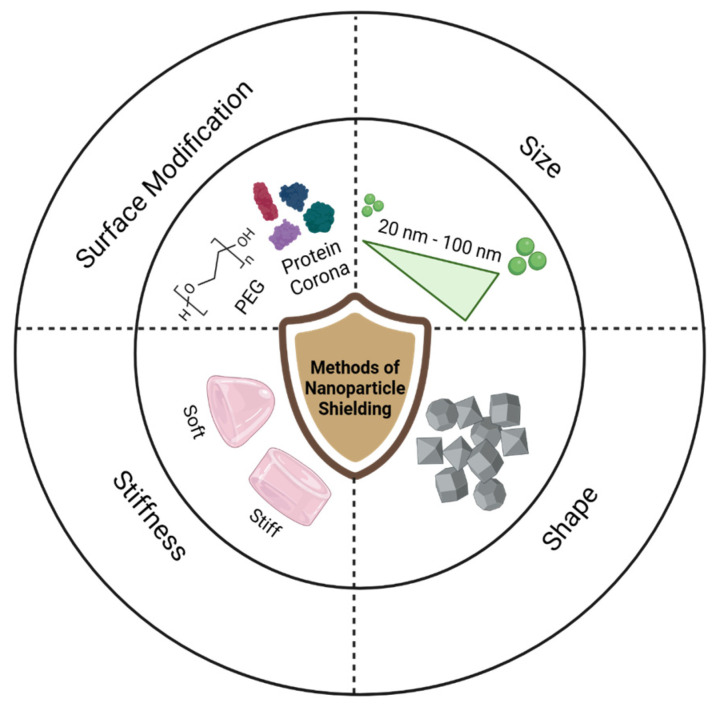
Graphical representation of common methods of nanoparticle shielding, including surface coatings of PEG and proteins, size alteration, shape differentiation, and stiffness scaling.

Fine-tuning the physicochemical properties of nanoparticles, including surface modifications such as poly (ethylene glycol) (PEG) coating or protein corona alterations, along with adjustments to size, shape, and stiffness, contributes to immune evasion of nanoparticles, namely nanoparticle shielding, as illustrated in Figure 2. These shielding methods alter the way opsonins adsorb to the particle surface and can lead to reduced immune cell recognition.

### 2.1. Shielding Through Surface Chemistry

Researchers have tried to solve these ubiquitous issues in nanomedicine by altering nanoparticle surface coatings, size, shape, and mechanical properties [30]. Currently, the gold standard and prevailing method of creating stealth nanoparticles that can evade immune detection comes from the PEG coatings on nanoparticles [22]. However, some studies indicate that prolonged exposure to PEG can create an anti-PEG immune response, making it ineffective for long-term cancer treatments [31]. In addition, it has been cited as creating an accelerated blood clearance (ABC) phenomenon, wherein second-dose PEG particles are cleared rapidly [32].

Other surface modifications include creating a customized protein corona to surround the nanoparticle as it enters the body [33]. This allows the researcher to manually conjugate the proteins that immune cells first identify to a particle. However, this method has many logistical issues, so researchers must consider details such as ligand density, orientation, 3D structure, and the fallibility of non-covalent surface bonds [34], which can be used to attach proteins to a particle surface. These include electrostatic interactions and metal-mediated interactions. However, these non-covalent methods are easily altered by changes in environmental pH and ionic strength and are easily reversible. For the purpose of drug delivery, this can be a negative consequence of non-covalent bonds, as the ligand could detach before the nanoparticle reaches its target. This method, while creative, cannot cover the full spectrum of surface proteins that would prevent particles from immune recognition [35].

### 2.2. Shielding Through Size

Another method of trying to avoid immune clearance involves editing particle size. According to Hirn et al., particle accumulation in the liver increases with size [36]. However, this relationship is not simply linear. According to Jang et al., who completed a study on particles ranging from 20 to 100 nanometers in size, the middle-of-the-road 60 nanometer diameter particles experienced the largest immune response [37]. A study conducted by Kulkarni et al. found no distinction in uptake to the liver or spleen with respect to nanoparticle size alone [38,39]. While it is generally accepted that particles under 200 nanometers in diameter are best for drug delivery, disagreeing results indicate that surface coating, shape, and composition are more immediately relevant to immune uptake.

### 2.3. Shielding Through Shape

Nanoparticle shape can also heavily influence immune cell uptake. While the word “particle” typically elicits an understanding of a sphere, nanoparticles come in many shapes, including not just spheres but also rods, ellipses, cubes, and various other 3D polygons [40]. It has been shown that macrophages are able to recognize the shape of their targets [41]. Macrophages are even understood to have preferential uptake for rod-shaped nanoparticles due to their larger surface area for the macrophage to grab and recognize. After rods, spheres show the highest capability for general uptake, followed by cylinders and cubes [42]. Repeated studies have shown that spheres are the best shape for evading immune capture [43].

### 2.4. Shielding Through Elastic Modulus

Beyond size, shape, and surface, the mechanical properties of a nanoparticle also heavily influence its immune clearance [44]. Merkel et al. show that altering a hydrogel microparticle’s elastic modulus can affect its circulation time [45]. When testing the elastic moduli of particles between 8 kPa and 64 kPa, it was found that particles with the lowest modulus of elasticity had the longest distribution half-life. This is made more interesting by the fact that red blood cells have a Young’s modulus of around 8 kPa [46], indicating that the particles most similar to the body’s naturally circulating cells were able to stay in circulation longer. This phenomenon is also material-dependent. Wang et al. found that an increase in stiffness increases lipid bilayer membrane permeability in hydrophilic nanoparticles, while a decrease in stiffness benefits the permeability of hydrophobic particles.

## 3. Methods of Nanoparticle Targeting

**Figure 3:**
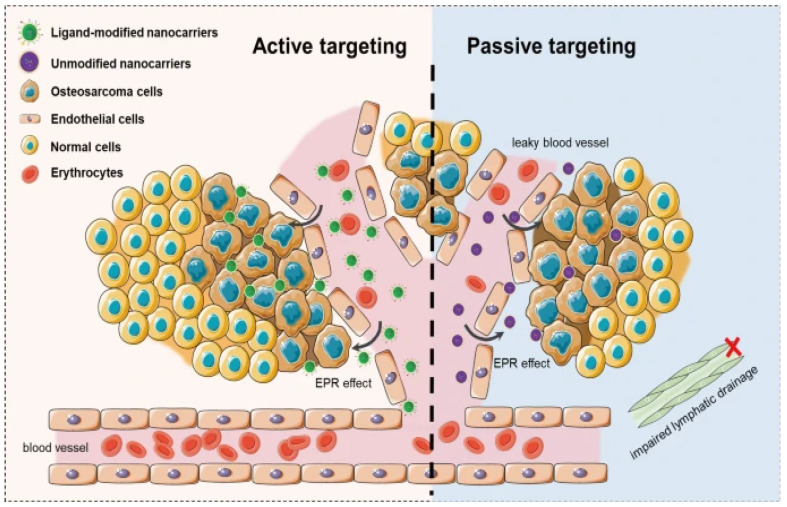
Representative scheme of active targeting and passive targeting in nanoparticle-based delivery systems [47].

The word “targeting” is used plenty in nanomedicine, but is not strictly accurate; neither active nor passive targeting creates a tumor-seeking particle that can locate and navigate its way to a target. Instead, both methods rely on the enhanced permeation and retention effect, utilizing the leaky vasculature created by tumor angiogenesis, as shown in Figure 3. What makes active targeting “active” are nanoparticle surface modifications that, should the particle coincidentally find its way to cancer, should enhance delivery efficiency through ligand binding.

### 3.1. Targeting Through Ligand Conjugation

Most cancer cell targeting in nanomedicine relies on nanoparticles with ligand binding [48]. This is known as active targeting, wherein attaching a cell-specific ligand and/or antibody to the surface of a nanoparticle can increase the chances of a nanoparticle making contact with its intended target [48,49]. Specifically, attaching antibodies to a nanoparticle surface creates a powerful dual therapeutic approach of delivering chemotherapeutics and initiating immunotherapeutic strategies [50]. However, this method does not directly pull nanoparticles toward their destination but instead increases the odds of intratumoral nanoparticle uptake, mainly via prolonged circulation [27]. While this method works well in vitro, it often fails in vivo due to the oxymoronic nature of tumor vasculature; tumors have “leaky” vasculature, but this can usually increase interstitial pressure, which limits outward bulk flow, the means by which particles enter tumors [51].

### 3.2. Intratumoral Injections

The standard nanoparticle delivery method in vivo includes injecting particles directly into the bloodstream. In mouse studies, often the first level of animal studies in nanomedicine, this means injecting nanoparticles intravenously [52]. Without direct targeting, though, particles can get removed from the bloodstream and filtered out by the reticuloendothelial system without ever reaching their target. In order to combat this, scientists have developed a method of intratumoral injection [53]. Patently, this is where nanoparticles are injected directly into the tumor, leaving no need to go through the treacherous circulation system. This method works well enough for solid tumors but is not feasible for many hematological cancers where there is no solid tumor.

### 3.3. The Consonant Nature of Nanoparticle Shielding and Targeting

When sending particles through the body’s circulation system to combat blood cancers, the ultimate goal is to allow the nanoparticle the maximum chance to reach a tumor cell within the vasculature. In order to reach this goal, the nanoparticles must stay in circulation long enough to be able to pass by a cancer cell. The two foremost goals of targeting blood cancers with nanomedicine, particle shielding and targeting, are thus intertwined and are not truly separate objectives.

## 4. Introduction to Biomimicry

**Figure 4:**
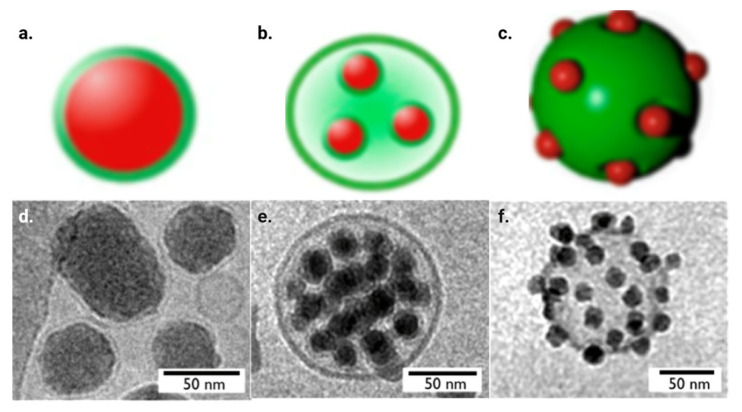
Digital representation images of (**a**) lipid bilayer-wrapped nanoparticles, (**b**) cellular Trojan horses and (**c**) cellular backpacks, as well as cryogenic transmission electron microscopy (TEM) images of (**d**) lipid bilayer-wrapped nanoparticles, (**e**) cellular Trojan horses and (**f**) cellular backpacks using silica particles [55].

Biomimicry, or the imitation of naturally occurring phenomena or systems for novel purposes, is a rising branch of nanomedicine [1]. In nanomedicine, nanoparticles most often try to mimic cells, as they are not likely to cause alarm within the immune system [54]. Biomimicry in nanomedicine gained fame in 2011 when Hu et al. wrapped polymeric nanoparticles in red blood cell membranes and found that it improved circulation half-life compared to PEG-coated nanoparticles [22]. Since then, the field has taken off, incorporating not just cell-wrapping as a form of biomimicry but also cellular Trojan horses and cellular backpacks, as demonstrated in Figure 4.

### 4.1. Cell Membrane Wrapping

The original form of biomimicry in nanomedicine was wrapping nanoparticles in red blood cell membranes [22]. This method is effective because it allows for all the complex surface receptors on the cell membrane to be used for nanoparticle shielding without the need to try to replicate each ligand. In addition, isolating cells within a patient to then wrap nanoparticles with those cells can eliminate an immunogenic response as the body recognizes its own cells. Cells are first lysed hypotonically to remove the intracellular components for membrane wrapping. Then, membranes are extruded through films with pores of ~400 nanometers, which reshapes the loose membranes into spheres for wrapping. In order to get the nanoparticles into the now-emptied cell membranes, nanoparticles and membranes are extruded together, which pushes the nanoparticles into the membrane while keeping the overall shape. Alternate reconstruction methods of cell membrane-coated nanoparticles include joint sonication as well as microfluidic electroporation [56]. Excess cells and unwrapped nanoparticles can then be isolated and removed through centrifugation. Cryogenic TEM images can confirm that particles are wrapped in membranes, as shown in Figure 4d.

### 4.2. Cellular Trojan Horses

In wrapping with cell membranes, one needs first to empty all the intracellular components. This is not necessary for all biomimetic applications. For cellular Trojan horses, many nanoparticles can be purposefully loaded into cells to be carried to a destination [54,57]. Just like the Greeks used a wooden horse to sneak past enemy lines, nanoparticles can use cells to cross cellular barriers and infiltrate tumor armies. Monocytes and macrophages are often used for this purpose, as they have an inherent phagocytic ability that allows for the easy uptake of nanoparticles through just a 24 h incubation. In this process, the nanoparticles are taken into the cell in vacuoles and then dispersed in the cytoplasm, which allows for an easy exit once distributed in vivo. In essence, scientists have taken a challenge in nanomedicine, i.e., how easily immune cells uptake nanoparticles and turn them into a research boon. Cryogenic TEM imaging can also confirm that the cells have taken up nanoparticles, as shown in Figure 4e.

### 4.3. Cellular Backpacks

Cellular backpacks, or cellular patches, are similar to Trojan horses in that they use a lot of nanoparticles for one cell. For backpacks, though, the nanoparticles are conjugated to the cell’s exterior [58]. Another similarity to cellular Trojan horses is that the methodology requires simple incubation, with an added linker/cytokine to conjugate the nanoparticles to the cell surface. T-cells or mesenchymal stem cells are typically used, as they are naturally recruited to tumors and circulating tumor cells [54]. Cryogenic TEM images can show nanoparticles on the surface of cells, as shown in Figure 4f.

## 5. Biomimicry for Prolonged Circulation

In one of the first studies conducted on cell biomimicry for nanomedicine, it was found that at both 24 and 48 h, the RBC-coated nanoparticles had more considerable retention within the blood compared to PEG-coated nanoparticles, at 29% and 16%, to 11% and 2%, respectively [22]. Since then, researchers have expanded on their method and used cell biomimicry to prolong the circulation time for nanoparticles.

### 5.1. Cell Membrane Wrapping for Prolonged Circulation

Since that initial study, many different cell types have been used to wrap nanoparticles. Advances in the field have even been made to use hybrid cell membranes for wrapping by fusing two or more different membrane types to gain both benefits [59]. The emergence of hybrid cell membrane-coated nanoparticles represents a significant advancement in biomimetic technology. Chen et al. describe how these systems combine the synthetic versatility of artificial nanoparticles with the biological functionality of natural cell membranes, creating platforms that can effectively evade immune clearance while maintaining targeted therapeutic delivery [60]. Jiang et al. found that their cancer cell/red blood cell hybrid membrane-coated nanoparticles had a blood retention of about 15%, while their bare nanoparticles only had a retention rate of about 2%. These results were measured through fluorescence-labeled nanoparticles and verified through a biodistribution study. If the dye had leaked out of the cell-wrapped particles, kidney absorption would have increased over time, as fluorescent dyes are filtered through the kidney. However, liver fluorescence increased over time, meaning the dye stayed in the particles, leading to accurate results. This was a great verification of the study, elucidating how hybrid cell membrane wrapping can be used to prolong nanoparticle circulation time.

### 5.2. Cellular Trojan Horses for Prolonged Circulation

**Figure 5:**
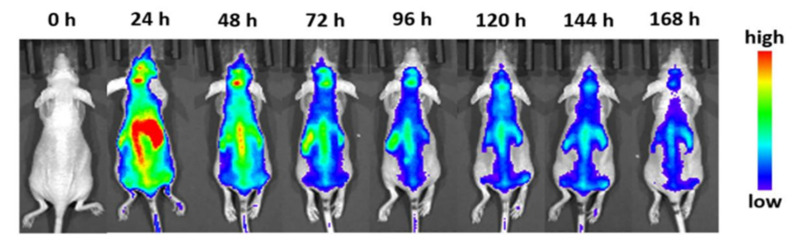
Fluorescence imaging of whole mice injected with macrophage exosome cellular Trojan horses at the selected time points [61].

In 2022, Wu et al. incubated nanocarriers with macrophage exosomes to form cellular Trojan horses to cross the blood-brain barrier [61]. In this study, they found that the in vivo fluorescent signal was greatest after 12 h, meaning that not only were the particles able to stay in the mouse for a good amount of time, but that the cell-internalized particles were able to gather together, as shown in Figure 5. In fact, it took up to 7 days for the nanocarriers to leave the system, indicating excellent retention and little immune clearance.

### 5.3. Cellular Backpacks for Prolonged Circulation

One of the foremost areas for the use of cellular backpacks is in drug delivery. Klyacho et al. determined that polymer patches could piggyback off macrophages, leading to prolonged drug delivery [62]. This study found that their antioxidant of choice, catalase, could stay in the bloodstream for over 54 h longer than catalase alone, indicating a successful inhibition of immune clearance. The cell backpacks also protect the catalase from proteinases in the blood, confirming synthetic nanocarriers as an overall drug delivery tool.

## 6. Biomimicry for Cancer Targeting

### 6.1. Homotypic Targeting

One challenge with conventional nanoparticle surface modifications for enhancing cancer targeting is their dependence on random interactions between the nanoparticles and cancer cells. However, cell biomimicry is uniquely adaptable for targeting cancer cells as it can utilize homotypic targeting—the inherent ability of cells to preferentially find and bind to their own cell type [63,64]. This is particularly unique to cancer cells, as they have unusually robust adhesive interactions [65]. Cancer cells use this abnormal adhesion to form circulating tumor cell aggregates, the foundation of metastasis, making cancer more dangerous and a larger target for drug delivery [66]. By using cancer cell membranes for cellular biomimicry in nanomedicine, scientists can utilize its benefits in prolonging circulation time and specifically target cancers lacking solid tumors.

### 6.2. Cell Membrane Wrapping for Cancer Targeting

**Figure 6:**
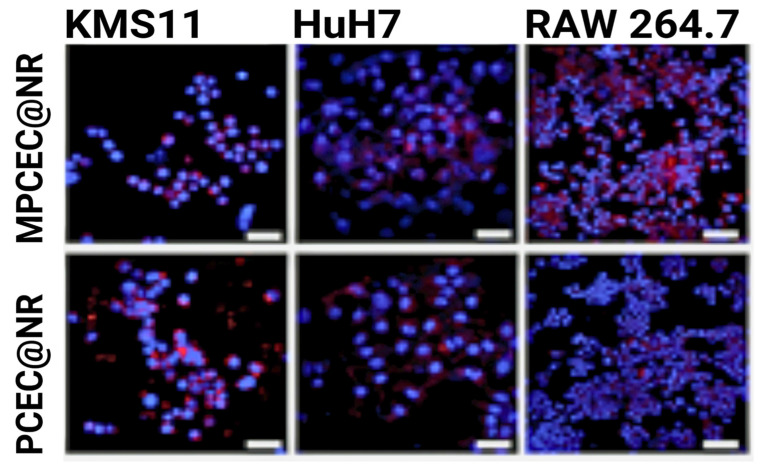
KMS11 cell membrane-wrapped nanoparticle internalization in three cell lines, where red indicates nanoparticle fluorescence and blue indicates cells [67].

In order to combat multiple myeloma, a common hematological cancer affecting the bone marrow, Qu et al. coated chemotherapeutic-loaded nanoparticles with multiple myeloma cell membranes [67]. When compared to non-membrane-coated particles, the cell-wrapped particles vastly outperformed in cancer cell internalization, beating them 18-fold. To verify the homotypic targeting, they also studied nanoparticle uptake in multiple cancer cell lines and found that the myeloma-wrapped particles exhibited the highest internalization in their own cell line, KMS11, as seen in Figure 6.

### 6.3. Cellular Trojan Horses for Cancer Targeting

Extracellular vesicles (EVs) are naturally occurring nanoparticles from cancer cells and play a role in cancer metastasis [68]. These EVs are especially prominent in hematological cancers, which rely heavily on vasculature travel for metastasis. Qiao et al. used EVs from HeLa and HT1080 cells as cellular Trojan horses [69]. They found that in vivo in mice with cancers stemming from both cell lines, each Trojan horse had preferential binding to its own cell type. In vitro, they also showed that HeLa Trojan horses created the lowest cell viability in HeLa cells, indicating possible decreased side effects and toxicity in vivo.

### 6.4. Cellular Backpacks for Cancer Targeting

Some biomimetic nanomedicines are two-fold, working by attaching one natural structure to another. Hu et al. used hematopoietic stem cells as their carrier, with platelets as the backpacks to carry antibodies for the inhibition of leukemia [70]. They showed over 25-fold greater antibody accumulation in bone marrow compared to antibody-decorated platelets alone. This stipulates that homotypic targeting is a powerful tool for delivering anticancer treatments throughout the vasculature. In an in vivo murine leukemia model, researchers showed that mice with the cellular backpack treatment had a 62.5% survival rate at day 50, while mice without the treatment succumbed by day 40, indicating that a human study could lead to increased patient survival rates.

## 7. Conclusions and Future Perspectives

The field of nanomedicine has advanced significantly since its start roughly 55 years ago, providing innovative solutions for cancer treatment. Targeting hematologic cancers presents unique challenges, particularly in prolonging nanoparticle circulation time and ensuring effective drug delivery to the bloodstream without off-target effects in the absence of a solid tumor to target. Biomimicry offers a promising approach to address these obstacles through strategies such as cell membrane wrapping, cellular Trojan horses, and cellular backpacks. Each of these techniques has demonstrated improved circulation time and specific targeting capabilities, paving the way for more efficient and less invasive therapies for hematologic cancers.

### 7.1. Possible Limitations

While the potential of biomimicry is clear, several areas require further exploration to fully realize its clinical applications. Current challenges include scalability for mass production, repressing possible immunogenic responses, and developing universally applicable methods for biomimetic nanoparticle synthesis. Moreover, while cell-derived biomimicry reduces immune clearance and enhances targeting, its safety profile, immunogenicity, and long-term effects must be comprehensively evaluated through rigorous preclinical and clinical trials. Despite these challenges, advancements in nanotechnology and bioengineering continue to bring us closer to overcoming these hurdles, offering the immense potential to revolutionize targeted cancer therapies.

### 7.2. The Future of Nanomedicine

Researchers are actively focusing on integrating hybrid biomimetic systems that combine multiple functionalities, such as prolonged circulation, specific targeting, and controlled drug release. Additionally, leveraging advancements in computational and pharmacokinetic modeling can optimize biomimetic design, predicting outcomes and enhancing the efficiency of nanoparticle development. As the field progresses, interdisciplinary collaborations between material scientists, biologists, and clinicians will be pivotal in translating these innovative technologies from theory to therapy. Ultimately, biomimicry represents a transformative frontier in nanomedicine, particularly for hematologic cancers, by harnessing the body’s own mechanisms to outsmart immune clearance and improve therapeutic outcomes. With continued research and technological advancements, biomimicry has the potential not only to overcome current challenges in nanomedicine but also to set new paradigms for personalized and effective cancer treatment.

## Data Availability

No new data were created or analyzed in this study.

## References

[1] Himel M.H., Sikder B., Ahmed T., Choudhury S.M. (2022). Biomimicry in nanotechnology: A comprehensive review. Nanoscale Adv..

[2] Krukemeyer M.G., Krenn V., Huebner F., Wagner W., Resch R. (2015). History and Possible Uses of Nanomedicine Based on Nanoparticles and Nanotechnological Progress. J. Nanomed. Nanotechnol..

[3] Zhou J., Kroll A.V., Holay M., Fang R.H., Zhang L. (2019). Biomimetic Nanotechnology toward Personalized Vaccines. Adv. Mater..

[4] Li J., Sharkey C.C., Huang D., King M.R. (2015). Nanobiotechnology for the Therapeutic Targeting of Cancer Cells in Blood. Cell. Mol. Bioeng..

[5] Alkhatib A.J. (2024). Nanomedicine for Targeted Drug Delivery Systems: A Mini-Review. Int. J. Nanotechnol. Allied Sci..

[6] Tsatsakis P.A.M., Vliora D.M., Paraskevi D.K., Kalkach-Aparicio D.M. (2024). Emerging Social Issues on Targeted Drug Delivery.

[7] Liu R., Luo C., Pang Z., Zhang J., Ruan S., Wu M., Wang L., Sun T., Li N., Han L. (2022). Advances of nanoparticles as drug delivery systems for disease diagnosis and treatment. Chin. Chem. Lett..

[8] Jain K.K. (2008). The Handbook of Nanomedicine.

[9] Wu X., Wang F., Yang X., Gong Y., Niu T., Chu B., Qu Y., Qian Z. (2024). Advances in Drug Delivery Systems for the Treatment of Acute Myeloid Leukemia. Small.

[10] Wang J., Sheng L., Lai Y., Xu Z. (2022). An overview on therapeutic efficacy and challenges of nanoparticles in blood cancer therapy. J. King Saud Univ.-Sci..

[11] Blood Cancer Statistics | LLS. https://www.lls.org/facts-and-statistics/facts-and-statistics-overview.

[12] Zheng Y., Sun Y., Yu X., Shao Y., Zhang P., Dai G., Fu J. (2016). Angiogenesis in Liquid Tumors: An In Vitro Assay for Leukemic-Cell-Induced Bone Marrow Angiogenesis. Adv. Heal. Mater..

[13] Davis A.S., Viera A.J., Mead M.D. (2014). Leukemia: An overview for primary care. Am. Fam. Physician.

[14] Mugnaini E.N., Ghosh N. (2016). Lymphoma. Prim. Care Clin. Off. Pr..

[15] Cowan A.J., Green D.J., Kwok M., Lee S., Coffey D.G., Holmberg L.A., Tuazon S., Gopal A.K., Libby E.N. (2022). Diagnosis and Management of Multiple Myeloma A Review. JAMA.

[16] Relapsed and Refractory Disease: What It Means for Blood Cancer Patients|Fox Chase Cancer Center-Philadelphia PA. https://www.foxchase.org/blog/relapsed-and-refractory-disease-what-it-means-for-blood-cancer-patients.

[17] Bringing Out the Big Guns Against Blood Cancer|NIH Intramural Research Program. https://irp.nih.gov/blog/post/2022/09/bringing-out-the-big-guns-against-blood-cancer.

[18] Powsner E.H., Harris J.C., Day E.S. (2021). Biomimetic Nanoparticles for the Treatment of Hematologic Malignancies. Adv. NanoBiomed Res..

[19] Dunleavy K., Wilson W.H. (2012). How I treat HIV-associated lymphoma. Blood.

[20] Chiang C.-L., Cheng M.-H., Lin C.-H. (2021). From Nanoparticles to Cancer Nanomedicine: Old Problems with New Solutions. Nanomaterials.

[21] Caracciolo G., Palchetti S., Colapicchioni V., Digiacomo L., Pozzi D., Capriotti A.L., La Barbera G., Laganà A. (2015). Stealth Effect of Biomolecular Corona on Nanoparticle Uptake by Immune Cells. Langmuir.

[22] Hu C.-M.J., Zhang L., Aryal S., Cheung C., Fang R.H., Zhang L. (2011). Erythrocyte membrane-camouflaged polymeric nanoparticles as a biomimetic delivery platform. Proc. Natl. Acad. Sci. USA.

[23] Mitchell M.J., Billingsley M.M., Haley R.M., Wechsler M.E., Peppas N.A., Langer R. (2020). Engineering precision nanoparticles for drug delivery. Nat. Rev. Drug Discov..

[24] The Entry of Nanoparticles into Solid Tumours|Nature Materials. https://www.nature.com/articles/s41563-019-0566-2.

[25] Estrella V., Chen T., Lloyd M., Wojtkowiak J., Cornnell H.H., Ibrahim-Hashim A., Bailey K., Balagurunathan Y., Rothberg J.M., Sloane B.F. (2013). Acidity Generated by the Tumor Microenvironment Drives Local Invasion. Cancer Res..

[26] Gwinn M.R., Vallyathan V. (2006). Nanoparticles: Health Effects—Pros and Cons. Environ. Heal. Perspect..

[27] Bae Y.H., Park K. (2011). Targeted drug delivery to tumors: Myths, reality and possibility. J. Control. Release.

[28] Chen S., Zhong Y., Fan W., Xiang J., Wang G., Zhou Q., Wang J., Geng Y., Sun R., Zhang Z. (2021). Enhanced tumour penetration and prolonged circulation in blood of polyzwitterion–drug conjugates with cell-membrane affinity. Nat. Biomed. Eng..

[29] Veiga N., Diesendruck Y., Peer D. (2023). Targeted nanomedicine: Lessons learned and future directions. J. Control. Release.

[30] Moghimi S.M., Wagner E. (2017). Nanoparticle Technology: Having Impact, but Needing Further Optimization. Mol. Ther..

[31] Knop K., Hoogenboom R., Fischer D., Schubert U.S. (2010). Poly(ethylene glycol) in Drug Delivery: Pros and Cons as Well as Potential Alternatives. Angew. Chem. Int. Ed..

[32] Zalba S., Hagen T.L.T., Burgui C., Garrido M.J. (2022). Stealth nanoparticles in oncology: Facing the PEG dilemma. J. Control. Release.

[33] Lin Z., Li S., Wu Q., Qu H., Shi X., Wang K., Tang C., Yin C. (2024). In situ customized apolipoprotein B48-enriched protein corona enhances oral gene delivery of chitosan-based nanoparticles. Biomaterials.

[34] Ly P.-D., Ly K.-N., Phan H.-L., Nguyen H.H.T., Duong V.-A. (2024). Recent advances in surface decoration of nanoparticles in drug delivery. Front. Nanotechnol..

[35] Li Z., Shan X., Chen Z., Gao N., Zeng W., Zeng X., Mei L. (2020). Applications of Surface Modification Technologies in Nanomedicine for Deep Tumor Penetration. Adv. Sci..

[36] Hirn S., Semmler-Behnke M., Schleh C., Wenk A., Lipka J., Schäffler M., Takenaka S., Möller W., Schmid G., Simon U. (2011). Particle size-dependent and surface charge-dependent biodistribution of gold nanoparticles after intravenous administration. Eur. J. Pharm. Biopharm..

[37] Kim S., Oh W.-K., Jeong Y.S., Hong J.-Y., Cho B.-R., Hahn J.-S., Jang J. (2011). Cytotoxicity of, and innate immune response to, size-controlled polypyrrole nanoparticles in mammalian cells. Biomaterials.

[38] Kulkarni S.A., Feng S.-S. (2013). Effects of Particle Size and Surface Modification on Cellular Uptake and Biodistribution of Polymeric Nanoparticles for Drug Delivery. Pharm. Res..

[39] Hoshyar N., Gray S., Han H., Bao G. (2016). The effect of nanoparticle size on in vivo pharmacokinetics and cellular interaction. Nanomedicine.

[40] Hadji H., Bouchemal K. (2022). Effect of micro- and nanoparticle shape on biological processes. J. Control. Release.

[41] Doshi N., Mitragotri S. (2010). Macrophages Recognize Size and Shape of Their Targets. PLoS ONE.

[42] Gratton S.E., Ropp P.A., Pohlhaus P.D., Luft J.C., Madden V.J., Napier M.E., DeSimone J.M. (2008). The effect of particle design on cellular internalization pathways. Proc. Natl. Acad. Sci. USA.

[43] Liu Y., Hardie J., Zhang X., Rotello V.M. (2017). Effects of engineered nanoparticles on the innate immune system. Semin. Immunol..

[44] Gurnani P., Sanchez-Cano C., Xandri-Monje H., Zhang J., Ellacott S.H., Mansfield E.D.H., Hartlieb M., Dallmann R., Perrier S. (2022). Probing the Effect of Rigidity on the Cellular Uptake of Core-Shell Nanoparticles: Stiffness Effects are Size Dependent. Small.

[45] Merkel T.J., Jones S.W., Herlihy K.P., Kersey F.R., Shields A.R., Napier M., Luft J.C., Wu H., Zamboni W.C., Wang A.Z. (2011). Using mechanobiological mimicry of red blood cells to extend circulation times of hydrogel microparticles. Proc. Natl. Acad. Sci. USA.

[46] Kumari S., Mehendale N., Roy T., Sen S., Mitra D., Paul D. (2024). Measuring red blood cell deformability and its heterogeneity using a fast microfluidic device. Cell Rep. Phys. Sci..

[47] Shi P., Cheng Z., Zhao K., Chen Y., Zhang A., Gan W., Zhang Y. (2023). Active targeting schemes for nano-drug delivery systems in osteosarcoma therapeutics. J. Nanobiotechnol..

[48] Tietjen G.T., Bracaglia L.G., Saltzman W.M., Pober J.S. (2018). Focus on Fundamentals: Achieving Effective Nanoparticle Targeting. Trends Mol. Med..

[49] Li R., Zheng K., Yuan C., Chen Z., Huang M. (2017). Be Active or Not: The Relative Contribution of Active and Passive Tumor Targeting of Nanomaterials. Nanotheranostics.

[50] Oroojalian F., Beygi M., Baradaran B., Mokhtarzadeh A., Shahbazi M.A. (2021). Immune Cell Membrane-Coated Biomimetic Nanoparticles for Targeted Cancer Therapy. Small.

[51] Thurber G.M., Schmidt M.M., Wittrup K.D. (2008). Antibody tumor penetration: Transport opposed by systemic and antigen-mediated clearance. Adv. Drug Deliv. Rev..

[52] Zhang X.D., Wu H.Y., Wu D., Wang Y.Y., Chang J.H., Zhai Z.B., Meng A.M., Liu P.X., Zhang L.A., Fan F.Y. (2010). Toxicologic effects of gold nanoparticles in vivo by different administration routes. Int. J. Nanomed..

[53] Holback H., Yeo Y. (2011). Intratumoral Drug Delivery with Nanoparticulate Carriers. Pharm. Res..

[54] Advances in Targeted Nanotherapeutics: From Bioconjugation to Biomimicry—PMC. https://pmc.ncbi.nlm.nih.gov/articles/PMC6879063/.

[55] Mousseau F., Oikonomou E.K., Baldim V., Mornet S., Berret J.-F. (2018). Nanoparticle-Lipid Interaction: Job Scattering Plots to Differentiate Vesicle Aggregation from Supported Lipid Bilayer Formation. Colloids Interfaces.

[56] Liu H., Su Y.-Y., Jiang X.-C., Gao J.-Q. (2022). Cell membrane-coated nanoparticles: A novel multifunctional biomimetic drug delivery system. Drug Deliv. Transl. Res..

[57] Choi M.-R., Stanton-Maxey K.J., Stanley J.K., Levin C.S., Bardhan R., Akin D., Badve S., Sturgis J., Robinson J.P., Bashir R. (2007). A Cellular Trojan Horse for Delivery of Therapeutic Nanoparticles into Tumors. Nano Lett..

[58] Stephan M.T., Moon J.J., Um S.H., Bershteyn A., Irvine D.J. (2010). Therapeutic cell engineering with surface-conjugated synthetic nanoparticles. Nat. Med..

[59] Jiang Q., Liu Y., Guo R., Yao X., Sung S., Pang Z., Yang W. (2019). Erythrocyte-cancer hybrid membrane-camouflaged melanin nanoparticles for enhancing photothermal therapy efficacy in tumors. Biomaterials.

[60] Chen H.-Y., Deng J., Wang Y., Wu C.-Q., Li X., Dai H.-W. (2020). Hybrid cell membrane-coated nanoparticles: A multifunctional biomimetic platform for cancer diagnosis and therapy. Acta Biomater..

[61] Wu T., Liu Y., Cao Y., Liu Z. (2022). Engineering Macrophage Exosome Disguised Biodegradable Nanoplatform for Enhanced Sonodynamic Therapy of Glioblastoma. Adv. Mater..

[62] Klyachko N.L., Polak R., Haney M.J., Zhao Y., Neto R.J.G., Hill M.C., Kabanov A.V., Cohen R.E., Rubner M.F., Batrakova E.V. (2017). Macrophages with cellular backpacks for targeted drug delivery to the brain. Biomaterials.

[63] Harris J.C., Scully M.A., Day E.S. (2019). Cancer Cell Membrane-Coated Nanoparticles for Cancer Management. Cancers.

[64] De Pasquale D., Marino A., Tapeinos C., Pucci C., Rocchiccioli S., Michelucci E., Finamore F., McDonnell L., Scarpellini A., Lauciello S. (2020). Homotypic targeting and drug delivery in glioblastoma cells through cell membrane-coated boron nitride nanotubes. Mater. Des..

[65] Janiszewska M., Primi M.C., Izard T. (2020). Cell adhesion in cancer: Beyond the migration of single cells. J. Biol. Chem..

[66] Sun H., Su J., Meng Q., Yin Q., Chen L., Gu W., Zhang P., Zhang Z., Yu H., Wang S. (2016). Cancer-Cell-Biomimetic Nanoparticles for Targeted Therapy of Homotypic Tumors. Adv. Mater..

[67] Qu Y., Chu B., Wei X., Chen Y., Yang Y., Hu D., Huang J., Wang F., Chen M., Zheng Y. (2021). Cancer-Cell-Biomimetic Nanoparticles for Targeted Therapy of Multiple Myeloma Based on Bone Marrow Homing. Adv. Mater..

[68] Cheng X., Henick B.S., Cheng K. (2024). Anticancer Therapy Targeting Cancer-Derived Extracellular Vesicles. ACS Nano.

[69] Qiao L., Hu S., Huang K., Su T., Li Z., Vandergriff A., Cores J., Dinh P.-U., Allen T., Shen D. (2020). Tumor cell-derived exosomes home to their cells of origin and can be used as Trojan horses to deliver cancer drugs. Theranostics.

[70] Hu Q., Sun W., Wang J., Ruan H., Zhang X., Ye Y., Shen S., Wang C., Lu W., Cheng K. (2018). Conjugation of haematopoietic stem cells and platelets decorated with anti-PD-1 antibodies augments anti-leukaemia efficacy. Nat. Biomed. Eng..

